# Low-dose intravenous immunoglobulin treatment for complex regional pain syndrome (LIPS): study protocol for a randomized controlled trial

**DOI:** 10.1186/1745-6215-15-404

**Published:** 2014-10-24

**Authors:** Andreas Goebel, Nicholas Shenker, Nick Padfield, Karim Shoukrey, Candida McCabe, Mick Serpell, Mark Sanders, Caroline Murphy, Amaka Ejibe, Holly Milligan, Joanna Kelly, Gareth Ambler

**Affiliations:** The University of Liverpool, Brownlow Hill, L69 7ZX Liverpool, UK; Addenbrookes Hospital, Hills Road, CB2 0QQ Cambridge, UK; Guy’s and St Thomas’ NHS Trust, Westminster Bridge Rd, SE1 7EH London, UK; University Hospital of Leicester NHS Trust, Gwendolen Road, LE5 9PW Leicester, UK; RNHRD and University West of England, Upper Borough Walls, BA1 1RL Bristol, UK; Gartnavel General Hospital, Great Western Road, G12 0YN Glasgow, UK; Norfolk and Norwich University NHS Trust, Colney Lane, NR4 7UY Norwich, UK; King’s Clinical Trials Unit at KHP, De Crespigny Park, SE5 8AF London, UK; University College London, Gower Street, WC1E 6BT London, UK

**Keywords:** CRPS, immunoglobulin, IVIg, pain

## Abstract

**Background:**

Longstanding complex regional pain syndrome (CRPS) is refractory to treatment with established analgesic drugs in most cases, and for many patients, alternative pain treatment approaches, such as with neuromodulation devices or rehabilitation methods, also do not work. The development of novel, effective treatment technologies is, therefore, important. There are preliminary data suggesting that low-dose immunoglobulin treatment may significantly reduce pain from longstanding CRPS.

**Methods/Design:**

LIPS is a multicentre (United Kingdom), double-blind, randomised parallel group, placebo-controlled trial, designed to evaluate the efficacy, safety, and tolerability of intravenous immunoglobulin (IVIg) 0.5 g/kg plus standard treatment, versus matched placebo plus standard treatment in 108 patients with longstanding complex regional pain syndrome. Participants with moderate or severe CRPS of between 1 and 5 years duration will be randomly allocated to receive IVIg 0.5 g/kg (IntratectTM 50 g/l solution for infusion) or matching placebo administered day 1 and day 22 after randomisation, followed by two optional doses of open-label medication on day 43 after randomisation and on day 64 after randomisation. The primary outcome is the patients’ pain intensity in the IVIG group compared with the placebo group, between 6 and 42 days after randomisation. The primary trial objective is to confirm the efficacy and confidently determine the effect size of the IVIG treatment technology in this group of patients.

**Trial registration:**

ISRCTN42179756 (Registered 28 June 13).

**Electronic supplementary material:**

The online version of this article (doi:10.1186/1745-6215-15-404) contains supplementary material, which is available to authorized users.

## Background

Complex regional pain syndrome (CRPS) is a post-traumatic pain in a limb, associated with sensory, motor, autonomic, skin and bone changes [1, 2]. CRPS can resolve spontaneously, but if a spontaneous resolution does not occur early, it is less likely to occur later. Many patients with CRPS have no effective method to relieve their ongoing pain [3]. Those patients with CRPS of moderate to severe pain intensity, the target group for this study, report on average a very poor quality of life, and they usually cannot work [4]. Immunoglobulin treatment for chronic pain is a novel technology [5]. In one of the first open trials, we found that low dose intravenous immunoglobulin (IVIg) may be effective in some patients with CRPS (for 11 participants: three had >70% pain relief, two had >25% but < 70%, and six had 0 to 25%, following a variable number of low-dose infusion repeats) [6]. We later showed that in one patient repeat treatments provided reproducible effects [7]. Recently, we confirmed in a UK single-centre crossover, randomised, placebo-controlled trial (RCT) [8], that a single, low dose (0.5 g/kg) infusion of IVIg significantly reduced pain in patients with CRPS (n = 13, pain intensity on a validated 11-point numeric rating scale (NRS) higher than 4 (NRS 0 = no pain, 10 = pain as bad as you can imagine [9]); these patients had a disease duration of 0.5 to 2.5 years. The treatment difference was 1.55 NRS points (95% CI: 1.29 to1.82, *P* <0.001). In a responder analysis (12 patients had received treatment), three patients had ≥50% less pain (4.5, 5 and 5 NRS points) after IVIg when compared with after saline treatment, and two patients had 2 and 2.5 NRS points less pain (29% and 31% less pain). One patient had 2 NRS points less pain (25% less pain) after saline compared with after IVIg treatment. The average effect duration was 5 weeks. There was also a significant overall reduction of CRPS-related, non-painful symptoms and, in responders, improved sleep and global improvement, with few adverse events (headaches and pain increases for <3 days). Post-infusion questionnaires showed successful blinding of patients and study doctors.

Recently we commenced a trial to explore whether subcutaneous immunoglobulin, in weekly self-administration at home over one year, would provide sustained pain relief in initial responders to 0.5 g/kg IVIg (ISRCTN63226217). We invited all five patients who experienced at least 2 NRS points less pain after IVIg in the earlier RCT. Of these patients, one declined participation, and a second patient unfortunately developed metastasizing colon cancer. Three patients participated. By August 2011, two patients, with disease durations of 6 and 5 years at study entry and baseline pain intensities of NRS 7 and 6 had experienced sustained pain reduction of >70% for 12 and 3.5 months, respectively. The third patient, who had had 31% relief in the RCT, showed no benefit. The two responding patients reported major improvement in their quality of life. EQ5D scores [10] improved from 0.26 and 0.30 at baseline to 0.66 and 0.65 at twelve and three months and reduced interference of their pain with daily functioning; Brief Pain Inventory [11] interference scores (pain interference = the impact of pain on activities of daily life) improved from 7.7 and 6.1 at baseline to 1.4 and 0 at twelve and three months.

The implication of the existing research for this trial is that the above evidence provides proof of concept for the efficacy of low-dose immunoglobulin treatment for patients with CRPS of moderate to severe pain intensity (msCRPS) in reducing pain, with an advantageous side-effect profile. These data also suggest that this treatment may improve quality of life and pain interference. Because the numbers of treated patients have been small, and most research was conducted in a single centre, it is now important to confirm these findings in a larger group of patients and across several centres, to gain confidence about both efficacy and affect size of this novel technology, and to demonstrate its generalizability.

The primary objective of this trial is to gain, within 44 months, both definite proof of the clinical efficacy and a more confident estimate of the effect size of low-dose IVIg treatment to reduce pain in patients with moderate or severe complex regional pain syndrome.

Secondary objectives are to achieve a better understanding of this technology including the following:

1. stability of effect with repeat administration;
2. factors predicting a beneficial response;
3. effects on additional outcome parameters including stimulus evoked pain, pain interference, quality of life, and short-term risk profile;
4. health economics evaluation; and
5. creation of a bank of biological samples for future complex regional pain syndrome research.

The primary outcome will be the average 24-h pain intensity over 37 days (to be completed on day 6 to day 42 after randomisation to record pain intensity during the previous 24 hours).

The number of study sites will be seven: all UK-based specialist pain clinics in secondary care (Liverpool, London, Bath, Glasgow, Norfolk and Norwich, Cambridge, Leicester).

The study population size will be 108 (54 in each study arm).

The study duration will be 44 months (from study set-up to analysis).

## Methods/Design

LIPS is a multicentre, randomised, double-blind, placebo-controlled, parallel group trial with an open extension. The parallel group design is an established research technique; the open extension is an optional trial element, where patients who have completed the parallel, blinded phase can request to receive one, or maximally two doses of IVIG ‘openly’, that is, assured that what they receive is IVIG (there is no placebo control). This extension is included to take account of service users’ preferences.

Blinding will be achieved by preparing both study drug and placebo (0.1% albumin in normal saline) solution into identical bottles. The albumin is added to achieve indistinguishable foaming and colour to the IVIg. Batch numbers and expiry dates for both the active and placebo drug will be indistinguishable.

For the time frame, study day 0 is defined as the day of randomisation, and a screening visit is scheduled maximally three weeks prior (day -21); patients then receive blinded infusions on days 1 and 22. Thereafter, in those who decide to receive open infusion on day 43 and day 64, pain diaries will be completed daily from day 43 to day 85 and then weekly for 9 weeks further to explore the duration of combined drug- and unspecific treatment effects (Additional file 1).

The sample size is 108 patients (54 patients per study arm). An interim analysis will be performed for futility and safety after half of patients have completed the trial. It will be suggested that the trial be stopped if there is a statistically significant difference between the groups in the ‘wrong’ direction at the 5% level (that is, one-sided test at the 2.5% level). This stopping rule will have a negligible effect on the type I error and power of the trial. There will no statistical stopping rule for efficacy although the DMC may suggest stopping the trial on the grounds of safety if there is an overwhelming positive effect of IVIg.

Clinical stopping rules will relate to unexpectedly poor recruitment and excessive withdrawals.

### Primary outcome measure

The primary outcome is the average 24-h pain intensity over 37 days, recorded in pain diary entries for the previous 24 hours and collected on days 6 to 42 (day 1 = day of first infusion). Consenting patients, who provide a mobile phone number, will be prompted automatically by SMS daily from day 2 to 42 to enter their pain intensity into their diary. In addition, return SMSs from the patient, with the daily NRS pain value, will be automatically saved as backup for the paper diary. The paper diary score will override the texted diary score; however, every effort will be made to resolve any discrepancies. In participating patients, an unexplained lack of a response over two or three days will prompt a phone call from the study nurse to confirm that there are no issues.

### Secondary and exploratory outcome measures

1. Secondary outcomes will be pain interference measured using the interference subscale of the Brief Pain Inventory [11], and quality of life measured using the Euroqol EQ-5D-5 L [12].
2. All other outcomes are exploratory
3. List of measures to be used (measurement times see Additional file 1)
4. Detailed daily - (three items: pain unpleasantness [13], average 24-h NRS pain intensity, last 24-h sleep quality [14]), and simplified weekly (weekly NRS pain intensity) pain diaries
5. Adverse events
6. Brief Pain Inventory- (diagram, worst pain intensity, and interference scales only) [11]
7. Concomitant medications
8. Concomitant therapies
9. Patient weight
10. Skin temperature measured with a surface thermometer
11. Limb volume measured with a water-bath technique
12. EQ-5D (5 Item) [12]
13. Expectations from treatment [15]
14. Functional items and fatigue suggested by and developed together with patient group (5 scales)
15. Patient Global Impression of change [16]
16. Hospital Anxiety and Depression Scale [17]
17. Health and social care utilisation
18. Limb exam recording Budapest CRPS signs, and any additional abnormalities on inspection, and sensory (cotton wool, pinprick, cold-fork) and motor (observation of active range) examination
19. McGill [18]
20. Quantitative Sensory Testing in 40 patients with stimulus-evoked pain, excepting thermosensitivities (only in three trial centres)
21. Sullivan’s Pain Catastrophising Scale [19]
22. Work interference (Stanford Presenteeism Scale) [20]
23. Neglect-like symptoms in CRPS [21]

### Definition of end of study

The end of the study will be the last participant’s final study contact at day 148 (for those who elect to receive open label infusions) or at day 64 (for those who elect not to receive open label infusion).

SAEs will be monitored for 21 days after final dose of IVIg or until resolution.

### Subject population

#### Inclusion criteria

1. Diagnosis of Complex Regional Pain Syndrome I or II according to Budapest research criteria (Additional file 2) [22]
2. Disease duration of 1 to 5 years and a mean pain intensity on an 11-point (0 to 10) Numeric Rating Scale (NRS) over the first seven daily entries after screening within a pre-defined range (see section 9 for details of pain thresholds for eligibility)
3. Failure to respond (poor efficacy or unacceptable side effects) to drugs recommended for the treatment of neuropathic pain [23], including pregabalin or gabapentin, tricyclic antidepressants, and mild and strong opioids (where not contraindicated or refused by the patient).
4. Previous specialised pain physiotherapy [24] (where not contraindicated or refused by the patient)
5. Willingness to confirm the use of adequate birth control while on the trial will be required in premenopausal women without evidence for an inability to become pregnant.
6. Willingness to not start any other treatment for CRPS during the parallel part of the trial
7. Age 18 years and above

#### Exclusion criteria

Any individuals meeting any of the following will be excluded from the study:

1. Other significant chronic pains, which in the view of the study doctor may make assessment of the pain arising from CRPS difficult.
2. If the patient recently started a new therapy for CRPS that, in the view of the study doctor, may change the patient’s pain level during the time of participation in the trial.
3. Unstable medical conditions.
4. Litigation. Patients in litigation will be excluded only if conclusion of that litigation is imminent during the course of the study.
5. If patient is pregnant or breastfeeding.
6. Complete IgA deficiency.
7. Rare contraindications to IVIg therapy as per summary of product characteristics (SmPC).
8. Receiving IVIg for other reasons.
9. Patient was previously enrolled in CRPS IVIg/SCIG trials.
10. Ongoing drug or alcohol abuse.
11. Psychiatric or mental health disorder that could, in the judgement of the site investigator, interfere with successful study participation.
12. Unwillingness or inability to complete daily diaries or an inability to understand the questionnaires being used.
13. Cancer other than basal cell carcinoma within the last 5 years. However, those patients who have received definitive treatment, such as curative surgery more than 6 months ago, with no known recurrence can be included.
14. A history of hypercoagulable or thrombophilic clotting abnormalities.
15. A history of thrombembolic events: ischaemic stroke, confirmed myocardial infarction, pulmonary embolism; deep venous thrombosis except where immobility related (for example, after injury or operation).
16. Unstable angina.
17. Renal failure or serum creatinine greater than 1.5 times the upper limit of normal at screening.
18. Any medical condition that, in the opinion of the investigator, would make it unsafe for the patient to participate or which would interfere with assessment of the outcome measures.
19. Participation in another interventional trial within 3 months of randomisation. Participation in non-interventional studies is not a reason for exclusion.

### Screening, recruitment and consent

Patients will be identified through clinics at each of the seven centres, which are all specialist pain/complex regional pain syndrome clinics in secondary care. Strategies will be implemented to maximise awareness of the trial in the patient population and increase referrals to the recruiting centres (see details of strategies to be employed in Additional file 3). Patients will be given the patient information sheet to read at least 24 hours before the screening visit, where they will give informed consent. At the screening visit, there will be an opportunity for the participants to ask questions of a member of staff trained in all trial procedures, as delegated by the PI. The Principal Investigator or a co-investigator at each site will ensure that the participants meet the inclusion and exclusion criteria at the point of screening.

Patients will be telephoned at least 8 days, and maximally 14 days, after screening to check pain diary scores and confirm eligibility to participate.

Visit 2 will be scheduled no earlier than 10 days, and no later than three weeks, after visit 1.

Screen failures may be rescreened ONLY where there is a short-term reason for ineligibility, such as non-availability for study visits due to planned holidays or an ongoing acute illness. Pain diary scores that make the patient ineligible cannot be considered a reason for rescreening.

A screening log will be kept at site to document details of patients invited to be screened for participation in the study. For patients who decline or are ineligible, this will document any reasons available for nonparticipation (where provided). The log will ensure potential participants are only approached once.

The original signed consent form will be retained in the investigator site file, with a copy in the participant’s hospital medical notes, and a copy provided to the participant. The participant will specifically consent to their GP being informed of their participation in the study.

The right to refuse to participate without giving reasons will be respected.

### Study medication

#### PlaceboM

Matching placebo infusions will be manufactured for the 5 g/100 ml and 10 g/200 ml IntratectTM IVIg infusion. These will be identical in appearance to the active infusions - they will be indistinguishable by colour and foaming of the infusion.

#### IntratectTM IVIg infusion

The experimental intervention is 0.5 g/kg IntratectTM IVIg infusion, in combination with ongoing normal standard treatment for complex regional pain syndrome.

For reported side effects of IntratectTM IVIg infusion, please refer to section 17.2 and the summary of product characteristics (Additional file 4).

Contraindications include

hypersensitivity to any of the components and

hypersensitivity to homologous immunoglobulin, especially in very rare cases of IgA deficiency, when the patient has antibodies against IgA.

#### Selection of doses for the trial

The study medication is being used within normal clinical doses.

#### Selection and timing of dose for each participant

Interventions will be available in 5 g/100 ml and 10 g/200 ml bottles IntratectTM IVIg infusion or matching placebo.

Each participant will be scheduled to receive infusions of active IntratectTM (0.5 g/kg) or matching placebo day 1 post-randomisation and day 22 post-randomisation. In exceptional circumstances, where a randomized patient does not attend for the first infusion on day 1, delay of the first infusion up to day 5 is acceptable. See in Additional file 1: table of events.

Data collection timelines remain the same, regardless of when infusions are received. Any patient who has not received his/her trial infusion by day 5, that is, before day 6, will be withdrawn and not given trial medication. All patients receiving any amount of trial infusion on days 1 to 5 will be included in the intention-to-treat analysis. All patients who receive ≥80% of the target dose on day 1 will be included in the per-protocol analysis. All patients will be offered open-label infusions of IntratectTM on day 43 and day 64 post-randomization. IntratectTM or placebo will be infused intravenously at an initial rate of not more than 1.4 ml/kg/hr for 30 minutes. If well tolerated, the rate of administration may be increased to a maximum of 2.5 ml/kg/hr for the remainder of the infusion. This is higher than the usual recommended rate of 1.9 ml/kg/hr in order to ensure that the entire infusion can be completed in a single day, and in view of the experience of clinicians, higher rates of infusion are generally well tolerated.

Infusion rate adjustments can be made if patients experience mild adverse clinical effects, reducing to 1.9 ml/kg/hr in the first instance and further if required, while aiming for sufficient time to complete the entire infusion in a single day.

### Identity and supply of investigational medicinal product

IntratectTM IVIg infusion 10 g/200 ml - Biotest UK IntratectTM IVIg infusion 5 g/100 ml - Biotest UK.

The trial medication is supplied as 100 ml and 200 ml bottles containing 5 g and 10 g IVIg. The volume prescribed per patient is weight determined (Table 1), with a target of 0.5 g/kg.

**Table 1:** **The dosing is based on the patient weight, clinicians should refer to this table before administering the study drug**

Weight range	Dose to be administered	Kits (100 ml) to be dispensed	Kits (200 ml) to be dispensed	Volume to be administered	Maximum hourly infusion rate
35.5 to 45.4 kg	20 g	-	2	400 ml	88 to 113 ml/hr
45.5 to 55.4 kg	25 g	1	2	500 ml	113 to 138 ml/hr
55.5 to 65.4 kg	30 g	-	3	600 ml	138 to 163 ml/hr
65.5 to 75.4 kg	35 g	1	3	700 ml	163 to 188 ml/hr
75.5 to 85.4 kg	40 g	-	4	800 ml	188 to 213 ml/hr
85.5 to 95.4 kg	45 g	1	4	900 ml	213 to 238 ml/hr
95.5 to 105.4 kg	50 g	-	5	1,000 ml	238 to 263 ml/hr
105.5 to 115.4 kg	55 g	1	5	1,100 ml	263 to 288 ml/hr
115.5 to 125.4 kg	60 g	-	6	1,200 ml	288 to 313 ml/hr
125.5 to 135.4 kg	65 g	1	6	1,300 ml	313 to 338 ml/hr
135.5 to 145.4 kg	70 g	-	7	1,400 ml	338 to 363 ml/hr
145.5 to 155.4 kg	75 g	1	7	1,500 ml	363 to 388 ml/hr
155.5 to165.4 kg	80 g	-	8	1,600 ml	388 to 413 ml/hr

### Packaging and labelling of investigational medicinal product

Investigational medicinal product will be supplied in individual 100 ml and 200 ml bottles, containing 5 g and 10 g IVIg, or 0.1% albumin in normal saline as a control. For the day 1 and day 22 infusions, each bottle will be blinded during dispensing by the study site pharmacy. Day 43 and Day 64 infusions will use unblinded bottles of IntratectTM 5 g/100 ml or 10 g/200 ml.

Packaging and labelling will be completed in accordance with Good Manufacturing Practice (GMP) Annex 13 requirements and GCP, by the Aseptic Manufacturing Pharmacy Unit (AMPU) at Royal Liverpool and Broadgreen Hospital, Liverpool.

#### Label design for primary and secondary packaging

**Figure 1:**
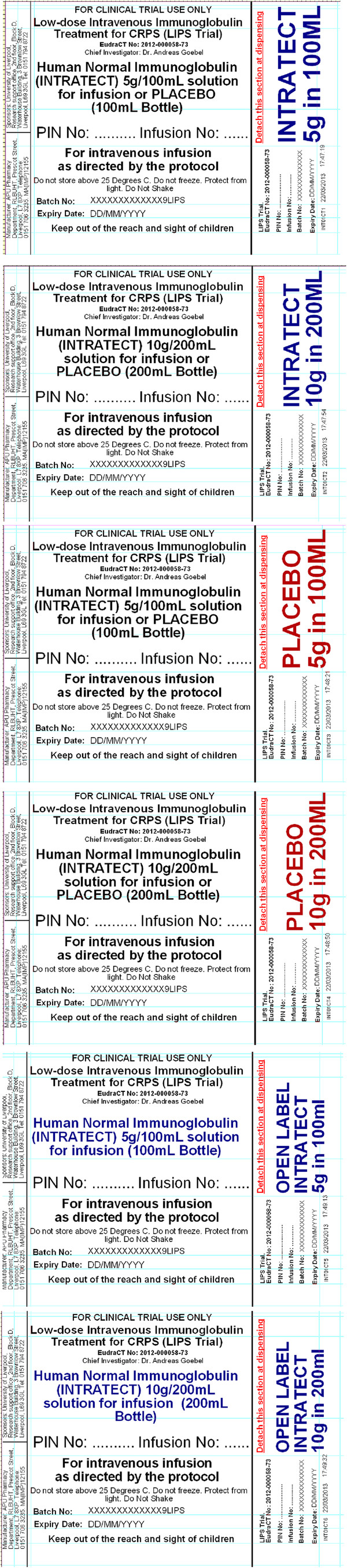
**Example labels of the primary container (bottle) and the secondary packing of the investigational medicinal product (IMP) and the placebo.**

Label designs will incorporate a structure that allows the IMP or placebo to remain blinded to clinical staff and participants. Both the primary container (bottle) and the secondary packing of the IMP and the placebo will be labelled in an identical manner (Figure 1).

All labels will carry a tear-off section that will be removed by the Pharmacy Departments at participating sites at the point of blinding and dispensing.

The 6 tear-off sections of labels that will be detached by the study site pharmacy department during dispensing are described as follows:

1. INTRATECT 10 g/200 ml,
2. INTRATECT 5 g/100 ml,
3. PLACEBO 5 g/100 ml,
4. PLACEBO 10 g/200 ml,
5. OPEN LABEL INTRATECT 5 g/100 ml, or
6. OPEN LABEL INTRATECT 10 g/200 ml.

### Prescription of investigational medicinal product

Study medication will be prescribed by an authorised study physician according to the protocol, using a trial-specific prescription. The volume to be dispensed per patient will be calculated according to patient weight (dosing-schedule Table 1) and the site pharmacist will dispense the required number of bottles. Medication will be dispensed according to local pharmacy practice. Bottles will contain 100 ml or 200 ml of IVIg or placebo. Participants will be informed of potential adverse reactions and advised to seek medical help and contact the research team, if required. Patients will carry cards with an emergency 24-hour code break number. Documentation of prescribing, dispensing and return of study medication shall be maintained for study records in the pharmacy file and reconciled with the investigator site file at end of study. A study-specific prescription must be submitted to pharmacy the day prior to the patient’s infusion. The pharmacy will have received an email from the randomisation service at the time of randomisation, which must be printed and filed with the dispensing records and which will be referred to by the dispensing pharmacist to decide whether to dispense active or placebo medication for the blinded infusions.

If the event that a 200-ml bottle is not available, it is permitted to dispense two 100-ml bottles, but where possible the preferred options are above. Where two 100-ml bottles are dispensed, the trial manager must be informed as the next shipment of drug to site may need to be adjusted as a result. The exception is where the IMP expiry is coming close and in the judgment of the site pharmacist, it makes sense to use up some expiring 100-ml bottles in preference to using 200-ml bottles with a later expiry. Ideally, this should be discussed with the trial manager in advance.

### Dispensing and distribution of investigational medicinal product

The study drug will be stored in a secure area with limited access within each local pharmacy according to the storage requirements documented on the clinical trial label prior to dispensing for each participant’s infusion visit. A temperature log will be maintained as per local pharmacy procedures.

Study medication will be distributed to the seven study site pharmacies by and from the Aseptic Manufacturing Pharmacy Unit (AMPU) at Royal Liverpool and Broadgreen Hospital, Liverpool. Study medication receipt will be recorded in the study pharmacy file. A study medication dispensing and return log will be maintained by the site pharmacies. Research staff will be instructed not to dispose of empty medication bottles, but to return these to pharmacy post-infusion.

Supplies of study medications dispensed on Day 1 and Day 22 post-randomization will be blinded by the study site pharmacy department by detaching the tear-off section from both the primary container (bottle) and the secondary packing. Dispensing records will be retained by the study site pharmacy department. For those who wish to receive open-label medication, either a single additional open-label dose will be given on Day 43 post-randomization, or additional open-label doses will be given on both Day 43 and Day 64 post-randomization (if patients wish to receive two open-label doses).

### Administration of investigational medicinal product

If centres prefer to run a slower infusion than described above, this will not be considered a protocol violation.

Patients may be offered paracetamol 1 g orally during the infusion, where clinically indicated. Patients are under continuous nurse observation during the infusion; in cases where no reduction of the infusion rate is required, the infusion duration for a participant of 75 to 85 kg body weight is about 4.5 hours.

In the event that patients do not receive their entire first infusion, either due to having to stop early because of time constraints arising from long infusion duration with a low rate, or because side effects are intolerable even with the lowest infusion rate, they should still be offered the second infusion, as patients often tolerate second infusions better. Details of the amount infused should be recorded in the medical notes and electronic Case Report Form (eCRF).

Where the infusion cannot be tolerated and the patient wishes to not receive additional infusion, the patient is withdrawn from further infusion, but follow-up data will be collected until the end of the study.

### Concomitant medications

All concomitant drug therapies received will be recorded at baseline and follow-up assessments.

Interactions include the following:

1. Live attenuated virus vaccines. Immunoglobulin administration may impair for a period of at least 6 weeks and up to 3 months the efficacy of live attenuated virus vaccines such as measles, rubella, mumps and varicella. After administration of this product, an interval of 3 months should elapse before vaccination with live attenuated virus vaccines. In the case of measles, this impairment may persist for up to 1 year. Therefore, patients receiving measles vaccine should have their antibody status checked.
2. Interference with serological testing. After injection of immunoglobulin the transitory rise of the various passively transferred antibodies in the patient’s blood may result in misleading positive results in serological testing. Passive transmission of antibodies to erythrocyte antigens, for example, A, B, and D, may interfere with some serological tests including the antiglobulin test (Coomb’s test).

Details of all other agents that might interact with IntratectTM can be found in the British National Formulary (BNF) (http://www.bnf.org/bnf/).

Study site investigators and patients will be provided with an emergency 24-hr unblinding contact number.

Participants should not receive any other investigational drugs or agents during their participation in the study.

Should patients experience a flare-up of their CRPS pain or a trial intervention-induced reduction in pain level, they may, in discussion with their PI (or a delegated experienced pain specialist), increase or reduce the dose of their current medications.

### Safety monitoring

The following blood tests will be done at baseline only. Additional blood monitoring is only required for the protocol in response to adverse events.

#### Routine haematology

Routine haematology includes the following:

1. white blood cell and differential count (eosinophils, basophils, neutrophils, lymphocytes and monocytes);
2. red blood cell and indices ( PCV, MCV, MCH , MCHC);
3. haemoglobin;
4. platelets;
5. serum IgA;
6. serum IgM; and
7. serum IgG.

#### Biochemistry

Routine biochemistry includes:

1. sodium;
2. potassium;
3. urea;
4. serum creatinine; and
5. ALT, AST, GGT, bilirubin.

#### Pregnancy

Pregnancy status will be verified using blood pregnancy testing at baseline for female patients of childbearing potential and urine pregnancy testing at visit 4 if patient is receiving open-label IntratectTM.

### Randomisation

#### Identification and randomisation of patients

A patient identification number will be allocated by registering the patient on the MACRO eCRF system after consent has been signed. The system will generate a unique identifier to be used throughout the study.

Patients will be allocated (on study day 0) to placebo or IVIg (ratio 1:1) by sites via an online system based at the King’s Clinical Trials Unit (King’s CTU), based at the Institute of Psychiatry. Allocation will be at the level of the individual patient via block randomisation with randomly varying block sizes, stratified by centre.

Only site staff authorised to request randomisation will receive passwords for the randomisation system. Requests for passwords are via the trial manager of the King’s CTU.

### Implementation procedures

Sites will be responsible for maintaining a baseline of ‘in date’ stock on shelf of both active and placebo IMPs.

Unblinded bottles for the open-label phase of the study will also be available and supplied to participating sites.

Once an eligible patient has provided written informed consent and completed the baseline assessments, he/she will be asked to complete pain diaries daily after the screening visit until the infusion visit. The study site investigator will contact the patient by telephone at an agreed time between 8 and 14 days after the screening visit and collect screening pain diary scores. If the patient is eligible on pain diary and blood results, an infusion visit will be scheduled.

Online randomisation will be requested by site one day prior to the infusion. Patients can only be randomised after the allocated study site nurse has confirmed by telephone a) that the patient is well, b) that the patient is willing and able to come to the infusion unit the next day, c) the details of the arranged transport, d) any anticipated problems from the patient’s perspective, and e) that the pharmacy will be able to dispense in good time.

The randomisation system will automatically generate two emails at the point of randomisation. The first will be sent to appropriate members of the study team, who are blinded to treatment allocation, and will just confirm that the patient has been randomised. The second will be sent to the dispensing pharmacy to inform them of the treatment allocation and will be copied to the eSMS emergency code break service, so they have the unblinding information available in the event of the need to unblind. A study-specific prescription will be completed (for dosing schedule, see Table 1) and sent to the pharmacy for dispensing. The dispensing pharmacist will refer to the randomisation email when the prescription is received. Any bottle of IMP in the appropriate trial arm can be selected for dispensing but if IMP is available with an earlier expiry this should be used in preference to IMP with a later expiry.

### Blinding

The trial will be double blinded. Blinding will be achieved by preparing both study drug and placebo (0.1% albumin in normal saline) solution into identical-looking bottles. Batch numbers and expiry dates for both active and placebo drug will be indistinguishable.

The IMP will be supplied directly to designated pharmacy contacts at participating sites. The tear-off section on the primary container (bottle) and the secondary packing will inform the dispensing site pharmacy of the true nature of contents (Active or Placebo). At dispensing, this section of label is removed to maintain blinding.

In the event of an urgent need to unblind treatment, the 24-hour emergency code break service must be contacted This should be the preferred route to code break, even in office hours when the site pharmacist is available, as there is then a full audit trail of the code break event. The site pharmacy will also be aware of the patient’s treatment allocation.

Unblinding should only occur where knowledge of the randomised treatment is needed for immediate patient care and cannot be delayed until the next working day when the study team can be contacted. Code breaks will not be routinely opened for participants who complete study treatment.

If a request for code break is received from a physician (for example, the patient’s general practitioner) outside the research team, Guy’s Medical Toxicology Unit (eSMS) will attempt to contact the research team to verify the request before the code is broken.

If the code is broken, details including patient study number, the date code break was performed, the person who broke the code, and the reason for the code break, shall be recorded by the emergency code break service and retained. The trial manager will be informed of the unblinding event. If clinically indicated, the participant will be withdrawn from the study medication.

Accidental unblindings will be dealt with on a case by case basis if and when they arise. The patient’s data should continue to be collected according to the visit schedule, even in the event of unblinding or withdrawal from study medication, unless the patient refuses.

### Study data

#### Trial database

An electronic Case Report Form (eCRF) will be created using the InferMed Macro system. This system is regulatory compliant (GCP, 21CRF11, EC Clinical Trial Directive). The eCRF will be created in collaboration with the trial statisticians and the investigators and will be maintained by the King’s Clinical Trials Unit. It will be hosted on a dedicated secure server within KCL.

Source data will be entered by authorised staff onto the eCRF with a full audit trail.

### Database passwords, data handling and confidentiality/format of records

Database access will be strictly restricted through passwords to the authorised research team.

Data will be handled, computerised and stored in accordance with the Data Protection Act, 1998. Participants will be identified on the study database using a unique code and initials. The investigator will maintain accurate patient records/results detailing observations on each patient enrolled. All participant contact/screening and recruitment data will be will be stored on spreadsheets within the recruiting NHS sites, which will have restricted access from password protected computers. Accrual data uploaded to the UKCRN portfolio database will be anonymised and collated by the Trial Manager to CLRN. No identifiable data will be entered on the eCRF or transferred to the coordinating CTU.

At the end of the study, essential documentation will be archived in accordance with sponsor and local requirements. The retention of study data will be the responsibility of the Chief Investigator.

### On-site/central monitoring

The Trial Manager will conduct on-site/central monitoring. The Data Manager/Statistician may identify data fields that should be checked against the source data during site monitoring visits, the specifics will be outlined in a Data Management standard operating procedure (SOP). Where there are data queries, the research nurses will be responsible for resolving the queries. The Trial Manager will review responses before closing the query.

### Statistical considerations

Data analysis will be performed by the study statistician at University College London, using a password-protected computer in a private office.

### Statistical analysis

A comprehensive statistical analysis plan will be developed and agreed upon with the trial’s oversight committees. Descriptive analysis (for example, summary statistics and plots) will be performed to investigate the distribution of the primary outcome, pain score, across participants.

#### Efficacy

##### Primary analysis

The primary outcome will be analysed using a mixed model to establish any difference between pain scores after IVIg and placebo. The stratification factor (centres) will be a fixed effect. The model will efficiently model the repeated measurements data. Modelling assumptions will be checked (for example, residuals). All analyses will be performed on an intention-to-treat basis. Every effort will be made to reduce loss to follow-up (using phone calls *etcetera*). Participants who do not contribute any outcome measurements for the primary outcome will be omitted from the analysis. Participants who provide any outcome data will be included. No imputation will be performed.

##### Secondary analysis

As a secondary analysis, we will calculate the proportion of patients in each arm who achieve 50% or 30% pain relief (based on the average pain level entered on day 6 to 42), compared to their baseline level of pain (the average pain level recorded during the first 7 days of the screening period). Using these proportions, we will calculate the number needed to treat (NNT) with IVIg, so that one additional patient will achieve 50% pain relief.

Possible changes in treatment effect over time and association between disease duration, psychological baseline measurements, allergy status, low baseline IgG plasma level, IgG increase, and treatment response, as well as any association between psychological measurements with the primary outcome, will be investigated using exploratory plots and regression models with interaction terms. Change in the McGill Pain Questionnaire (Short Form) descriptor terms [25] limb temperature and QST changes before-after IVIg/placebo treatments on affected/contra lateral sides, pain interference and quality of life (QoL) outcomes will be investigated using either standard regression models or mixed models. In those who decide to receive both open infusions, and who have at least 30% or 2 NRS points average pain relief from 6 to 20 days after their last open infusion as compared with baseline, the time between the last open infusion and the first period with average weekly pain equalling or exceeding baseline -1NRS point) is calculated as the IVIg effect duration. As the study ends on day 148 (12 weeks after the second open infusion), later effects will not be recorded.

### Safety

An interim analysis will be performed for futility and safety after half of patients have completed the trial. The trial may be stopped if there is a statistically significant difference between the groups at the 5% level (two-sided test), and if with the statistician remaining blinded, the unblinded DMC recognizes that the effect is in the ‘wrong’ direction. This stopping rule will only have a minor effect on the type I error and power of the trial.

There will no statistical stopping rule for efficacy although the data monitoring committee (DMC) may suggest stopping the trial on the grounds of safety if there is an overwhelming positive effect of IVIg.

All causes of withdrawal from randomised treatment will be reported at days 22 and 43 post- randomisation. The prevalence of adverse events and reactions will be reported descriptively at 22 and 43 days post-randomisation. For patients an open-label infusion, AE’s reported from 43 to 85 days post-randomisation will be tabulated separately for reports rather than being reported with blinded AE’s.

### Sample size calculation

The sample size was calculated as follows: 122 participants are required to detect a difference in pain score of 1.2 using a two-sample t-test assuming 5% statistical significance, 85% power and a common standard deviation of 2.2 (as in our previous study (8)). Assuming 10% loss to follow-up and 5% noncompliance increases, this number increases to 152 participants. We actually intend to collect 37 measurements of pain intensity (the primary outcome) per participant and analyse the outcome using a mixed-effects regression model.

Thus we can reduce this sample size based on these extra measurements. The correlation among a patient’s measures is assumed to be 0.7 (from our previous study), and hence, the multiplying factor is as follows:


Therefore, the total required sample size is 152 × 0.71 = 108 participants [26].

### Compliance and withdrawal

#### Subject compliance

Compliance will be measured by attendance at infusion visits on day 1 and day 22 and tolerance of entire prescribed infusion.

#### Treatment cessation

Patients who develop an unexpected new condition precluding further participation will be withdrawn from receiving further infusions, but will continue to complete daily pain diaries and asked to attend for collection of other outcome data (intention to treat). If patients do not tolerate blinded infusion 1, they may still be administered blinded infusion 2 if both the patient and clinician are agreeable.

#### Withdrawal of participants

Study drug must be discontinued for the following:

1. the participant decides he/she no longer wishes to continue;
2. withdrawal is recommended by the Investigator or another clinician (for example, intercurrent illness during course of study or side effects from study drug); or
3. the trial is terminated at the request of the DMC.

Patients will be discontinued for the following:

1. they are randomized, but never receive any drug (that is, the first infusion is never started - this is also termed ‘non-compliance’) or
2. they do not provide any values for the day 6 to 43 outcome pain diary data (this is also termed ‘missing data’).

Participants have the right to withdraw from the study at any time and for any reason without providing a reason. The investigator also has the right to withdraw participants from the study if they consider that it is in the best interests of the participant. Should a participant decide to withdraw from the study, he/she will be asked to volunteer a reason for withdrawal but are at liberty not to state a reason.

Should a participant withdraw from study drug only, efforts will be made to continue to obtain follow-up data with the permission of the patient. Subjects who withdraw from treatment early will be encouraged to return to the study site for follow-up until day 43, providing that consent is not withdrawn.

### Data monitoring, quality control and quality assurance

#### Discontinuation rules

The trial may be prematurely discontinued on the basis of predefined stopping criteria, or for other reasons given by the independent Data Monitoring and Ethics Committee, study sponsor, regulatory authority or ethics committee concerned.

#### Monitoring, quality control and assurance

The LIPS protocol has been developed with extensive review by clinicians, statisticians, and patient groups.

The LIPS Trial Coordination Centre will be based in the King’s Clinical Trials Unit in the Department of Biostatistics at the Institute of Psychiatry, King’s College London (IoP/KCL). Day to day management of LIPS will be the responsibility of the Chief Investigator and the Trial Manager. The Coordinating Centre will arrange meetings of the Trial Management Group (TMG) and Trial Steering Committee (TSC) and will coordinate independent Data Monitoring Committee (DMC) meetings.

The TMG will comprise Dr Andreas Goebel (Chair), the Trial Manager, the Data Manager, the Trial Statistician, and the manager of King’s Clinical Trials Unit. The TMG will arrange telephone conferences and provide monthly recruitment email updates during recruitment and status reports, and 6 monthly thereafter. The TMG will organise a meeting for all PIs (and for key staff working on the study) to sign the protocol, and to agree on the content and undergo training on SOPs before the start of recruitment. A second investigators’ meeting will be held at the end of the study to review the results. The TMG will also meet face-to-face every 6 months in Liverpool or London. The TMG will report to the Trial Steering Committee (TSC). All PIs will be kept informed of TSC and DMC advice and consulted by e-mail or teleconference as required.

The TSC will consist of an independent chair, two independent members, a site investigator, a patient representative, a nonvoting member, and the Chief Investigator; the names are detailed on page 4 of this protocol. The TSC will be responsible for approving the trial protocol and overseeing the conduct of the study, including advising on continuing or stopping the study in the light of advice from the DMEC. Meetings of the TSC will be held at least annually.

Membership of the DMEC will comprise an Independent Chair; an independent specialist with interest in neuropathic pain analgesic trials, and an independent statistician. The DMC will have access to the unblinded data and will monitor the progress of the trial in terms of safety and ethical issues. The DMC may advise the TSC to continue or to stop the trial according to pre-agreed upon stopping rules.

The Principal Investigators will be responsible for the day-to-day study conduct at site. This includes establishing and carrying out the trial at his/her centre in accordance with international, national and local law and regulations and GCP. They will ensure that all site specific documentation is complete and correct; and that all staff involved in the trial are compliant with Trust, GMC and other relevant regulations, and that they are appropriately trained in those aspects of GCP relevant to their role in the study whilst being familiar with the trial protocol.Principle Investigators are also responsible for managing recruitment on target and collecting and submitting accrual and outcome data in a timely manner; responding in timely fashion to requests from the Trial Coordinating Centre for information; providing and responding promptly to SAE and suspected unexpected serious adverse reaction (SUSARs) reports; agreeing to monitoring and audit visits as required.

Central and site monitoring of study conduct and data collected will be performed by the Trial Manager at the KCTU on behalf of the Sponsor. Full details will be documented in a monitoring plan, agreed upon with the study sponsor. The main areas of focus will include consent, serious adverse events, essential documents, and drug accountability and management. All monitoring findings will be reported and followed up with appropriate persons in a timely manner.

The study may also be subject to audit or inspection by the University of Liverpool or the Walton NHS Trust under their remit as co-sponsors, or by MHRA or other regulatory bodies to ensure adherence to GCP and regulatory requirements.

### Direct access to source data and documents

The investigators agree to provide full access to all source data, study data and materials to the trust research governance department, ethics committee, regulatory authority and trial manager for purposes of monitoring, audit or inspection.

### Pharmacovigilence

#### Expected adverse reactions

Adverse event reporting will be in compliance with GCP. Most adverse drug reactions that occur in this study, whether serious or not, will be expected treatment-related side effects as IVIg has a well-established side-effect profile.

IntratectTM can cause adverse reactions such as chills, headache, fever, vomiting, allergic reactions, nausea, arthralgia, low blood pressure and mild back pain, which may occur occasionally.

Rarely, human normal immunoglobulins may cause a sudden fall in blood pressure, and in isolated cases, anaphylactic shock, even when the patient has shown no hypersensitivity to previous administration.

Cases of reversible aseptic meningitis, isolated cases of reversible haemolytic anaemia/haemolysis, and rare cases of transient cutaneous reactions have been observed with human normal immunoglobulin.

Increase in serum creatinine level and/or acute renal failure have been observed.

Very rarely, thromboembolic reactions, such as myocardial infarction, stroke, pulmonary embolism, and deep vein thromboses have occurred.

Details of further spontaneously reported adverse reactions include the following:

1. cardiac disorders: angina pectoris (very rare);
2. general disorders and administrations site conditions: rigors (very rare);
3. immune system disorders: anaphylactoid shock (very rare), hypersensitivity (very rare);
4. investigations: blood pressure decreased (very rare);
5. musculoskeletal and connective tissue disorders: back pain (very rare);
6. respiratory, thoracic and mediastinal disorders: dyspnoea NOS (very rare); or
7. vascular disorders: shock (very rare).

The adverse events reported above are expected in the sense that they are possible known side-effects of the study medication, but all reported instances of both serious and non-serious adverse events will be reported in this study.

During the trial, investigators will be made aware of any updates to the summary of product characteristics (SPC) but the protocol need not be amended every time there is a change unless it directly affects the study conduct. The source of accurate information regarding the active medication must always be the SPC and not the study protocol, and the above information is provided to reflect the situation at study start only.

### Protocol specifications

#### For purposes of this protocol

1. Any serious adverse events will be recorded throughout the duration of the trial until 21 days after cessation of study drug or until resolution.
2. Non-serious adverse events will be recorded throughout duration of trial until 21 days after cessation of study drug.
3. Serious adverse events exclude any pre-planned hospitalisations not associated with clinical deterioration.

### Recording and reporting serious adverse events or reactions

All adverse events and all serious adverse events should be recorded. Depending on the nature of the event, the reporting procedures below should be followed. Any questions concerning adverse event recording/reporting should be directed to the Trial Manager in the first instance.

#### Non-serious adverse events

All non-serious adverse events will be recorded on the study CRF. Severity of all AEs will be graded on a three-point scale of intensity (mild, moderate, or severe):

Mild: Discomfort is noticed, but there is no disruption of normal daily activities.

Moderate: Discomfort is sufficient to reduce or affect normal daily activities.

Severe: Discomfort is incapacitating, with inability to work or to perform normal daily activities.

Relation of an AE to treatment should be assessed by the investigator/delegate (must be a clinician) on-site. Investigators will be responsible for managing all adverse events according to local protocols, as the study drug is already licensed for use in other indications.

#### Serious Adverse Event/Reaction (SAE/SAR, including SUSARs)

All SAEs, SARs and SUSARs shall be recorded and reported on the serious adverse event form to the Chief Investigator/delegate within 24 hours of learning of its occurrence. The initial report can be made by completing the serious adverse event form, and faxing or emailing into the King’s CTU. A record of this notification (including date of notification) must be clearly documented to provide an audit trail. In the case of incomplete information at the time of initial reporting, all appropriate information should be provided as follow-up as soon as this becomes available.

Relationship of the SAE to the treatment should be assessed by the investigator/delegate (must be a clinician) at that site, as should the expected or unexpected nature of any serious adverse reactions. As this is a blinded trial involving a placebo and active drug, seriousness, causality and expectedness should be evaluated as though the patient was on the active drug.

All SUSAR-reporting responsibilities to MHRA will be that of the Sponsor, with the support of the Kings CTU. The Sponsor will report SUSARs (Suspected Unexpected Serious Adverse Reactions) and other SARs to the regulatory authority (MHRA).

The Chief Investigator will report to the relevant ethics committees, with the support of the Kings CTU. Reporting timelines are as follow:

SUSARs that are fatal or life-threatening must be reported no later than 7 days after the sponsor is first aware of the reaction. Any additional relevant information must be reported within a further 8 days.

SUSARs that are not fatal or life-threatening must be reported within 15 days of the sponsor first becoming aware of the reaction.

The Chief Investigator will provide an annual report of all SARs (expected and unexpected), and SAEs which will be distributed to the Sponsor, MHRA and the REC.

As this is a blinded study, cases that are considered SUSARs would have to be unblinded to the King’s CTU manager prior to reporting to the Sponsor and main REC. Only those events occurring among patients on the active drug (unless thought to be due to the excipient in the placebo) should be considered SUSARs.

All investigators will be informed of all SAE’s assessed as fulfilling criteria as a SUSAR (that is, possibly, probably or definitely related to the study intervention and unexpected per the SPC) on a case-by-case basis. This will be regardless of medication administered in order to avoid the risk of inadvertently unblinding investigators, unless this information is needed for medical management of patients. Therefore, occasions may arise where a potential SUSAR is unblinded and if the patient is taking placebo, the MHRA would not need to be informed, and the investigators will not be made aware of this information. All reports to PIs will refer to events fulfilling criteria as a ‘potential SUSAR’ and only the KCTU and Sponsor will be aware of the events reported onward to the MHRA in an expedited manner.

The Chief Investigator will ensure University of Liverpool as lead sponsor is notified of any potential SUSARs.

*The Trial coordinating centre MUST be informed of all SAEs or SUSARs within 24 hours of learning of its occurrence. A record of this notification (including date of notification and acknowledgement of receipt from the KCTU) must be clearly documented to provide an audit trail.

The KCTU will onward report to the CI and Sponsor in compliance with regulatory requirements.

### Pregnancy

Should a trial participant become pregnant during the trial, she will be immediately withdrawn from study treatment, and the pregnancy will be followed up until outcome. The need to unblind will be considered on a case-by-case basis. Pregnancy will be reported as a serious adverse event. Data collection at the planned scheduled follow-up timeline must continue, unless the patient is unwilling to provide further data.

### Research blood samples

Research blood samples will be requested at baseline and day 43. Patients not consenting to provide research blood samples may still be randomised into the study. Patients only willing to provide research blood samples on a single occasion will just have the first sample taken.

On consenting patients, 30 ml of blood will be collected in a gel tube and centrifuged at 2,000 G for 10 minutes according to local policy (no specific centrifuge protocol is required). Serum must then be pipetted or poured into 10-ml aliquots and stored frozen in a -20 or -80 freezer. Each aliquot must be labelled with the patients study PIN, initials, date of birth and date of sample collection. Details of sample collection must be entered on the eCRF system.

The blood samples will be used to examine serum antibodies, mediators or substances in patients with complex regional pain syndrome. Samples will be stored and examined for 30 years.

On a periodic basis, the study monitor will arrange for a shipment of dry ice to be delivered to the study site and for samples to be shipped via courier to the University of Liverpool on the same day. Shipment forms will be provided to sites by the study monitor.

### Ethics and regulatory issues

The conduct of this study will be in accordance with the recommendations for physicians involved in research on human subjects adopted by the 18th World Medical Assembly, Helsinki 1964 and later revisions.

Information sheets will be provided to all eligible subjects and written informed consent obtained prior to any study procedures. Participants will be provided with a copy of the completed consent form for their records.

Favourable ethical opinion and MHRA Clinical Trial Authorisation has been obtained along with local approvals including site specific assessment and trust research and development approval. The participating sites have also signed a Clinical Trial Agreement (CTAg) with the study sponsor. The Trial Coordination Centre at the King’s Clinical Trials Unit required a written copy of local approval documentation and a copy of the signed CTAg before initiating each centre and accepting participants into the study.

Approval has been granted from the NRES Committee East of England - Hatfield.

The REC Reference Number is 12/EE/0164.

A detailed table showing all substantial amendments and what was changed is detailed in Additional file 5.

Local research and development approval has been granted from all infusion sites: Liverpool (The Walton Centre NHS Foundation Trust), Bath (Royal National Hospital for Rheumatic Disease NHS Foundation trust), Cambridge (Cambridge University Hospitals Foundation Trust at Addenbrookes), Glasgow (Greater Glasgow and Clyde Trust at Gartnavel), London (Guys and St Thomas’ NHS Foundation Trust), Norwich (Norfolk and Norwich University NHS Foundation Trust) and Leicester (University Hospitals of Leicester NHS Foundation Trust).

### Finance and insurance

NIHR EME is the main funder of this study. Biotest UK Ltd will provide active study medication free of charge and some funds. The Pain Relief Foundation, Liverpool, has provided additional support funding to the study.

The participating NHS Trusts have liability for clinical negligence that harms individuals towards whom they have a duty of care. NHS indemnity covers NHS staff and medical academic staff with honorary contracts conducting the trial. University insurance covers research staff who have their substantive contracts with the University for Potential Liability for issues arising from negligence in study design. There are no arrangements for non-negligent compensation.

### Publication policy

The data will be the property of the co-sponsors. Publication will be the responsibility of the Chief Investigator. Results from the study will be submitted for publication by the investigators only, in international medical journals. All manuscripts, abstracts or other modes of presentation will be reviewed by the TSC and DMC prior to submission. No reference will be made to any particular study subject. Results of the study will also be reported to the Sponsor and Funder in the required format.

Participants will be informed about their treatment allocation at the end of the study, along with a summary of the results, once the primary paper has been accepted for publication.

## Discussion

This protocol will allow us to determine if low dose intravenous immunoglobulin is an effective treatment for complex regional pain syndrome. We hope to have all 108 patients recruited by 30 November 2015 with write-up being completed by 31 July 2016.

## Trial status

Current Status: Open

Closure Date: 30 November 2015

Global Sample Size: 108

## Electronic supplementary material

Additional file 1: **Table of events - summary of study procedures.** It provides a comprehensive overview of the study procedures, the visit dates are shown along the top column, the left side column shows procedures and the crosses in the table indicate which visit they should be done on. Scheduled telephone calls are also shown on this table. (DOCX 61 KB)

Additional file 2: **Research Diagnostic Criteria (the “Budapest Criteria”) for Complex Regional Pain Syndrome.** It shows the research diagnostic criteria (Budapest) for Complex Regional Pain Syndrome, this is the criteria that patient’s illness must meet to be enrolled into the trial. (DOCX 14 KB)

Additional file 3: **Recruitment Strategy.** A table that summarises the various recruitment strategies utilised in this study given information on where, who would approach and how they then enrol. (DOCX 13 KB)

Additional file 4: **Intratect Summary of Product Characteristics.** A web link to a page that provides full details of the infusion drug. (DOCX 11 KB)

Additional file 5: **Summary of substantial amendments.** A table that details each substantial amendment that has been submitted for this protocol and what exactly was changed during that amendment. (DOCX 14 KB)

## Abbreviations

AE: adverse event
AR: adverse reaction
CRPS: complex regional pain syndrome
CTU: (King’s) Clinical Trials Unit, King’s College London (also KCTU)
DMC: data monitoring committee
eCRF: electronic Case Report Form
EME: efficacy and mechanism evaluation
GCP: good clinical practice
IVIg: intravenous immunoglobulin
IMP: investigational medicinal product
ISRCTN: international standardised randomised controlled trial number
MHRA: Medicines and Healthcare Products Regulatory Agency
msCRPS: patients with CRPS of moderate to severe pain intensity
MRIS: Medical Research Information Service
MRC: Medical Research Council
NICE: National Institute for Clinical Excellence
NIHR: National Institute for Health Research
NNT: number needed to treat
NRES: National Research Ethics Service
NRS: numeric rating scale
QoL: quality of life
REC: Research Ethics Committee
SAE: serious adverse event
SOP: standard operating procedure
SPC/SmPC: summary of product characteristics
SUSAR: suspected unexpected serious adverse reaction
TMG: Trial Management Group
TSC: Trial Steering Committee
UKCRN: UK Clinical Research Network.

## References

[1] Goebel A (2011). Complex regional pain syndrome in adults. Rheumatology (Oxford).

[2] Marinus J, Moseley GL, Birklein F, Baron R, Maihofner C, Kingery WS, van Hilten JJ (2011). Clinical features and pathophysiology of complex regional pain syndrome. Lancet Neurol.

[3] Forouzanfar T, Koke AJ, van Kleef M, Weber WE (2002). Treatment of complex regional pain syndrome type I. Eur J Pain.

[4] Kemler MA, Furnee CA (2002). Economic evaluation of spinal cord stimulation for chronic reflex sympathetic dystrophy. Neurology.

[5] Goebel A (2010). Immunoglobulin responsive chronic pain. J Clin Immunol.

[6] Goebel A, Netal S, Schedel R, Sprotte G (2002). Human pooled immunoglobulin in the treatment of chronic pain syndromes. Pain Med.

[7] Goebel A, Stock M, Deacon R, Sprotte G, Vincent A (2005). Intravenous immunoglobulin response and evidence for pathogenic antibodies in a case of complex regional pain syndrome 1. Ann Neurol.

[8] Goebel A, Baranowski AP, Maurer K, Ghiai A, McCabe C, Ambler G (2010). Intravenous immunoglobulin treatment of complex regional pain syndrome: a randomized trial. Ann Intern Med.

[9] Dworkin RH, Turk DC, Farrar JT, Haythornthwaite JA, Jensen MP, Katz NP, Kerns RD, Stucki G, Allen RR, Bellamy N, Carr DB, Chandler J, Cowan P, Dionne R, Galer BS, Hertz S, Jadad AR, Kramer LD, Manning DC, Martin S, McCormick CG, McDermott MP, McGrath P, Quessy S, Rappaport BA, Robbins W, Robinson JP, Rothman M, Royal MA, Simon L (2005). Core outcome measures for chronic pain clinical trials: IMMPACT recommendations. Pain.

[10] Brooks R (1996). EuroQol: the current state of play. Health Policy.

[11] Tan G, Jensen MP, Thornby JI, Shanti BF (2004). Validation of the brief pain inventory for chronic nonmalignant pain. J Pain.

[12] Herdman M, Gudex C, Lloyd A, Janssen M, Kind P, Parkin D, Bonsel G, Badia X (2011). Development and preliminary testing of the new five-level version of EQ-5D (EQ-5D-5 L). Qual Life Res.

[13] Dworkin RH, Turk DC, McDermott MP, Peirce-Sandner S, Burke LB, Cowan P, Farrar JT, Hertz S, Raja SN, Rappaport BA, Rauschkolb C, Sampaio C (2009). Interpreting the clinical importance of group differences in chronic pain clinical trials: IMMPACT recommendations. Pain.

[14] Cappelleri JC, Bushmakin AG, McDermott AM, Sadosky AB, Petrie CD, Martin S (2009). Psychometric properties of a single-item scale to assess sleep quality among individuals with fibromyalgia. Health Qual Life Outcomes.

[15] Wasan AD, Kong J, Pham LD, Kaptchuk TJ, Edwards R, Gollub RL (2010). The impact of placebo, psychopathology, and expectations on the response to acupuncture needling in patients with chronic low back pain. J Pain.

[16] Farrar JT, Young JP, LaMoreaux L, Werth JL, Poole RM (2001). Clinical importance of changes in chronic pain intensity measured on an 11-point numerical pain rating scale. Pain.

[17] Zigmond AS, Snaith RP (1983). The hospital anxiety and depression scale. Acta Psychiatr Scand.

[18] Dworkin RH, Turk DC, Revicki DA, Harding G, Coyne KS, Peirce-Sandner S, Bhagwat D, Everton D, Burke LB, Cowan P, Farrar JT, Hertz S, Max MB, Rappaport BA, Melzack R (2009). Development and initial validation of an expanded and revised version of the short-form McGill pain questionnaire (SF-MPQ-2). Pain.

[19] Sullivan LJ, Bishop SR, Pivik J (1995). The pain catastrophizing scale: development and validation. Psychologial Assessment.

[20] Koopman C, Pelletier KR, Murray JF, Sharda CE, Berger ML, Turpin RS, Hackleman P, Gibson P, Holmes DM, Bendel T (2002). Stanford presenteeism scale: health status and employee productivity. J Occup Environ Med.

[21] Frettlöh J, Hüppe M, Maier C (2006). Severity and specificity of neglect-like symptoms in patients with complex regional pain syndrome (CRPS) compared to chronic limb pain of other origins. Pain.

[22] Harden RN, Bruehl S, Stanton-Hicks M, Wilson PR (2007). Proposed new diagnostic criteria for complex regional pain syndrome. Pain Med.

[23] Dworkin RH, O’Connor AB, Backonja M, Farrar JT, Finnerup NB, Jensen TS, Kalso EA, Loeser JD, Miaskowski C, Nurmikko TJ, Portenoy RK, Rice AS, Stacey BR, Treede RD, Turk DC, Wallace MS (2007). Pharmacologic management of neuropathic pain: evidence-based recommendations. Pain.

[24] Oerlemans HM, Oostendorp RA, de Boo T, Goris RJ (1999). Pain and reduced mobility in complex regional pain syndrome I: outcome of a prospective randomised controlled clinical trial of adjuvant physical therapy versus occupational therapy. Pain.

[25] Melzack R (1987). The short-form McGill pain questionnaire. Pain.

[26] Machin D, Campbell MJ, Tan SB, Tan SH (2008). Sample size tables.

