# Supplementary material for: Low-dose intravenous immunoglobulin treatment for complex regional pain syndrome (LIPS): study protocol for a randomized controlled trial
Source: Trials. 2014 Oct 24;15:404. doi: 10.1186/1745-6215-15-404 (PMC4226877; doi:10.1186/1745-6215-15-404)
Supplement: Supplementary file 1 — Additional file 1: Table of events - summary of study procedures. It provides a comprehensive overview of the study procedures, the visit dates are shown along the top column, the left side column shows procedures and the crosses in the table indicate which visit they should be done on. Scheduled telephone calls are also shown on this table. (DOCX 61 KB) [file 13063_2013_2280_MOESM1_ESM.docx]

**Additional file 1:** Table of events - summary of study procedures

|  |  |  | **Day 0** | **Day 1** | **Day 2**  (+ up to 3 day) | **Day 5**  (+/- 2  days) | **Day 22** (+/-  1 day) | **Day 23** (+ up to 3 days) | **Day 26**  (+/- 2  days) | **Day 43** (+/- 1 day) | **Day 64** (+/-  1 day) | **Day 85** (+ up to 3 days) | **Day 148**  (+/- 1 day) |  |
| --- | --- | --- | --- | --- | --- | --- | --- | --- | --- | --- | --- | --- | --- | --- |
| **Study Week/month** | **Visit 1**  Screen  (Day - 21 to Day - | Telephone to confirm eligibility  (Day -11  to Day -1) | **Randomisation** | **Visit 2**  (1st blinded infusion) | **Telephone** | **Telephone** | **Visit 3**  (2nd blinded infusion) | **Telephone** | **Telephone** | **Visit 4**  (Obligatory, with optional 1st open infusion) | **Visit 5**  (This visit is only for 2nd open infusion) | **Telephone** | **Telephone End of Trial** | **Withdrawal** |
| **Registration/Demographics** | X |  |  |  |  |  |  |  |  |  |  |  |  |  |
| **Informed Consent** | X |  |  |  |  |  |  |  |  |  |  |  |  |  |
| **Eligibility form** | X |  |  |  |  |  |  |  |  |  |  |  |  |  |
| **Randomisation form** |  |  | X |  |  |  |  |  |  |  |  |  |  |  |
| **Medical History** | X |  |  |  |  |  |  |  |  |  |  |  |  |  |
| **CRPS History** | X |  |  |  |  |  |  |  |  |  |  |  |  |  |
| **Limb Exam** | X |  |  | X |  |  | X |  |  | X |  |  |  |  |
| **Limb temperature, limb volume** | X |  |  |  |  |  |  |  |  | X |  |  |  |  |
| **Safety bloods** (U&E,FBC,serum-Ig,LFT) | X |  |  |  |  |  |  |  |  |  |  |  |  |  |
| **Pregnancy test (beta HCG)** | X |  |  |  |  |  |  |  |  |  |  |  |  |  |
| **Pregnancy test (urine)** |  |  |  |  |  |  |  |  |  | X |  |  |  |  |
| **Screening pain diaries**  (Average 24h pain intensity only)  ^=day of collection back from patient, *=day issued to patient ) | * | X (over phone) |  | ^ |  |  |  |  |  |  |  |  |  |  |
| **Detailed (blind) diary, weeks 1,2,3** (average pain intensity, pain unpleasantness, sleep quality) (^=day of collection back from patient, *=day issued to patient)  (patients who consent will receive daily prompting texting reminders during days 2- 42) |  |  |  | * |  |  | ^ |  |  |  |  |  |  |  |
| **Detailed (blind) diary, weeks 4,5,6**  (average pain intensity, pain unpleasantness, sleep quality) (^=day of collection back from patient, *=day issued to patient)  (patients who consent will receive daily prompting texting reminders during days 2- |  |  |  |  |  |  | * |  |  | ^ |  |  |  |  |

|  | |  |  | **Day 0** | **Day 1** | **Day 2**  (+ up to 3 day) | **Day 5**  (+/- 2  days) | **Day 22** (+/-  1 day) | **Day 23** (+ up to 3 days) | **Day 26**  (+/- 2  days) | **Day 43** (+/- 1 day) | **Day 64** (+/-  1 day) | **Day 85** (+ up to 3 days) | **Day 148**  (+/- 1 day) |  |
| --- | --- | --- | --- | --- | --- | --- | --- | --- | --- | --- | --- | --- | --- | --- | --- |
| **Study Week/month** | | **Visit 1**  Screen  (Day - 21 to Day - | Telephone to confirm eligibility  (Day -11  to Day -1) | **Randomisati on** | **Visit 2**  (1st blinded infusion) | **Telephone** | **Telephone** | **Visit 3**  (2nd blinded infusion) | **Telephone** | **Telephone** | **Visit 4**  (Obligatory, with optional 1st open infusion) | **Visit 5**  (This visit is only for 2nd open infusion) | **Telephone** | **Telephone End of Trial** | **Withdrawal** |
| **Detailed (open) diary, weeks 7,8,9** (average pain intensity, pain unpleasantness, sleep quality) (^=day of collection back from patient, *=day issued to patient)  **For patients who receive open label infusion only.** | |  |  |  |  |  |  |  |  |  | * | ^ |  |  |  |
| **LIPS detailed (open) dairy, weeks 10, 11, 12**  (average pain intensity, pain unpleasantness, sleep quality) (^=day of collection back from patient, *=day issued to patient, ^1 diary returned using pre-paid envelope)  **For patients who receive open label infusion only** | |  |  |  |  |  |  |  |  |  |  | * | ^1 |  |  |
| **Simplified Pain Diaries** (weekly pain intensity scores only)  (^ = day diaries collected back from patient, *=day diaries issued to patient,^1 dairy returned using pre-paid envelope) | |  |  |  |  |  |  |  |  |  | * (patients who do not receive  open label infusion) | ^1  (patients who do not receive open infusion) | *  (patients who do receive open infusions) | ^1  (patients who do receive open infusions) | * |
| **Questionnaires:** | 1. Expectation from treatment |  |  |  | X |  |  |  |  |  |  |  |  |  |  |
|  | 2. EQ-5D-5L | x |  |  |  |  |  | x |  |  | x |  |  |  |  |
|  | 3. Mc Gill | x |  |  |  |  |  | x |  |  | x |  |  |  |  |
|  | 4. BPI | x |  |  |  |  |  | x |  |  | x |  |  |  |  |
|  | 5. HADs | x |  |  |  |  |  | x |  |  | x |  |  |  |  |
|  | 6. Pain Catastrophising | x |  |  |  |  |  | x |  |  | x |  |  |  |  |
|  | 7. Global impression of change |  |  |  |  |  |  | X |  |  | x | X |  | X |  |
|  | 8. Health/Social care utilisation | X |  |  |  |  |  |  |  |  |  |  |  |  |  |
|  | 9.Patient-developed measures | X |  |  |  |  |  | X |  |  | X |  |  |  |  |
|  | 10. Stanford Presenteeism | X |  |  |  |  |  |  |  |  | X |  |  |  |  |
|  | 11. Neglect-Like Symptoms | X |  |  |  |  |  |  |  |  | X |  |  |  |  |
| **Vital signs**  (pulse, blood pressure, before and after infusion) | |  |  |  | X |  |  | X |  |  | X | X |  |  |  |
| **Treatment Infusion Administration** | |  |  |  | X |  |  | X |  |  | X | X |  |  |  |
| **Research Bloods**  (30ml) | | x |  |  |  |  |  |  |  |  | X |  |  |  |  |

|  |  |  | **Day 0** | **Day 1** | **Day 2**  (+ up to 3 day) | **Day 5**  (+/- 2  days) | **Day 22** (+/-  1 day) | **Day 23** (+ up to 3 days) | **Day 26**  (+/- 2  days) | **Day 43** (+/- 1 day) | **Day 64** (+/-  1 day) | **Day 85** (+ up to 3 days) | **Day 148**  (+/- 1 day) |  |
| --- | --- | --- | --- | --- | --- | --- | --- | --- | --- | --- | --- | --- | --- | --- |
| **Study Week/month** | **Visit 1**  Screen  (Day - 21 to Day - | Telephone to confirm eligibility  (Day -11  to Day -1) | **Randomisati on** | **Visit 2**  (1st blinded infusion) | **Telephone** | **Telephone** | **Visit 3**  (2nd blinded infusion) | **Telephone** | **Telephone** | **Visit 4**  (Obligatory, with optional 1st open infusion) | **Visit 5**  (This visit is only for 2nd open infusion) | **Telephone** | **Telephone End of Trial** | **Withdrawal** |
| **Quantitative Sensory Testing (QST)**  (Subset of 40 patients only) | x |  |  | X  (if not done on visit 1) |  |  |  |  |  | X |  |  |  |  |
| **Concomitant & CRPS Pain Treatments Medications** | x | x |  | X | x |  | x |  |  | x | X |  | x | x |
| **Concomitant Therapies** | x | x |  | X | x |  | x |  |  | x | X |  | x | x |
| **Adverse Events Form** |  |  |  | X | x |  | x |  |  | x | X |  | x | x |
| **Patient Medication Guess** |  |  |  | X | x |  | x |  |  |  |  |  |  | x |
| **Physician Medication Guess** |  |  |  | X |  |  | x |  |  |  |  |  |  | x |
| **Research Nurse Medication Guess** |  |  |  | X |  |  | x |  |  |  |  |  |  | x |
| **Withdrawal Form** |  |  |  |  |  |  |  |  |  |  |  |  |  | x |

**Table Legend: McGill = McGill Pain Questionnaire (Short Form) pain descriptors; BPI = Brief Pain Inventory interference scores; HADS= Hospital Anxiety and Depression Scale; Catastrophising= Sullivan’s catastrophising scale; Global Impression = Patient Global Impression of Change Scale; Stanford Presenteeism Scale = Work interference ; LFT = Liver function test; FBC = Full blood count; Beta HCG = Beta Human chorionic gonadotropin**
