# Supplementary material for: Low-dose intravenous immunoglobulin treatment for complex regional pain syndrome (LIPS): study protocol for a randomized controlled trial
Source: Trials. 2014 Oct 24;15:404. doi: 10.1186/1745-6215-15-404 (PMC4226877; doi:10.1186/1745-6215-15-404)
Supplement: Supplementary file 2 — Additional file 2: Research Diagnostic Criteria (the “Budapest Criteria”) for Complex Regional Pain Syndrome. It shows the research diagnostic criteria (Budapest) for Complex Regional Pain Syndrome, this is the criteria that patient’s illness must meet to be enrolled into the trial. (DOCX 14 KB) [file 13063_2013_2280_MOESM2_ESM.docx]

Additional file 2 shows the research diagnostic criteria (Budapest) for Complex Regional Pain Syndrome, this is the criteria that patient’s illness must meet to be enrolled into the trial.

**Additional file 2**

**Research Diagnostic Criteria (the “Budapest Criteria”) for** Complex Regional Pain Syndrome

**General definition of the syndrome:**
Complex Regional Pain Syndrome describes an array of painful conditions that are characterized by a continuing (spontaneous and/or evoked) regional pain that is seemingly disproportionate in time or degree to the usual course of any known trauma or other lesion. The pain is regional (not in a specific nerve territory or dermatome) and usually has a distal predominance of abnormal sensory, motor, sudomotor, vasomotor, and/or trophic findings. The syndrome shows variable progression over time.

**To make the clinical diagnosis, the following criteria must be met:**

1. Continuing pain, which is disproportionate to any inciting event

2. Must report at least one symptom in all four following categories:

- Sensory: Reports of hyperesthesia and/or allodynia
- Vasomotor: Reports of temperature asymmetry and/or skin color changes and/or skin color asymmetry
- Sudomotor/Edema: Reports of edema and/or sweating changes and/or sweating asymmetry
- Motor/Trophic: Reports of decreased range of motion and/or motor dysfunction (weakness, tremor, dystonia) and/or trophic changes (hair, nail, skin)

3. Must display at least one sign at time of evaluation in two or more of the following categories:

- Sensory: Evidence of hyperalgesia (to pinprick) and/or allodynia (to light touch and/or temperature sensation and/or deep somatic pressure and/or joint movement)
- Vasomotor: Evidence of temperature asymmetry (>1 °C) and/or skin color changes and/or asymmetry
- Sudomotor/Edema: Evidence of edema and/or sweating changes and/or sweating asymmetry
- Motor/Trophic: Evidence of decreased range of motion and/or motor dysfunction (weakness, tremor, dystonia) and/or trophic changes (hair, nail, skin)

4. There is no other diagnosis that better explains the signs and symptoms
