# Supplementary material for: Low-dose intravenous immunoglobulin treatment for complex regional pain syndrome (LIPS): study protocol for a randomized controlled trial
Source: Trials. 2014 Oct 24;15:404. doi: 10.1186/1745-6215-15-404 (PMC4226877; doi:10.1186/1745-6215-15-404)
Supplement: Supplementary file 3 — Additional file 3: Recruitment Strategy. A table that summarises the various recruitment strategies utilised in this study given information on where, who would approach and how they then enrol. (DOCX 13 KB) [file 13063_2013_2280_MOESM3_ESM.docx]

Additional file 3 is a table that summarises the various recruitment strategies utilised in this study given information on where, who would approach and how they then enrol.

**Additional file 3**

Recruitment Strategy

|  | Method of approach | Ways in which patients can respond, in order to request for more info, or arrange screening, or decline | Where there is no patient response to the approach after 14 days, contact with |
| --- | --- | --- | --- |
| Approach through infusing centre | In clinic with Patient Infor-mation Sheet (PIS) | By phone | Reminder letter |
|  | Through invitation letter with PIS | Using a response slip or by phone | If no response within 14 days of the reminder letter: phone call |
| Approach through named  Patient Identifi-cation Centre (PIC) | In clinic with PIS | By phone or by response slip directly to the infusion centre (with notification to own doctor), or to the PIC, with request to forward contact details to the infusion centre | Reminder letter sent by (PIC) |
|  | Through invitation letter with PIS |  | If no response within 14 days of the reminder letter: phone call (by PIC doctor) |
| Approach through any UK doctor | Generally in clinic without PIS | Generally patients will directly confirm their interest to be contacted by one of the infusing centres, so that the doctor can then forward contact details to the infusion centre. | N/A |

**Table legend.** PIS=Patient Information sheet; N/A= Not applicable; PIC=Patient identification centres

Note: patients or doctors contacting one centre will be made aware of the closest centre to the patient’s home address.

Patients registered on the Complex Regional Pain Syndrome national registry will be approached by their nearest infusion centre through an invitation letter, as described in the first row
