# Supplementary material for: Low-dose intravenous immunoglobulin treatment for complex regional pain syndrome (LIPS): study protocol for a randomized controlled trial
Source: Trials. 2014 Oct 24;15:404. doi: 10.1186/1745-6215-15-404 (PMC4226877; doi:10.1186/1745-6215-15-404)
Supplement: Supplementary file 4 — Additional file 4: Intratect Summary of Product Characteristics. A web link to a page that provides full details of the infusion drug. (DOCX 11 KB) [file 13063_2013_2280_MOESM4_ESM.docx]

Additional file 4 is a web link to a page that provides full details of the infusion drug.

**Additional file 4**

Intratect Summary of Product Characteristics

http://www.medicines.org.uk/emc/medicine/23175/SPC/intratect/
