# Supplementary material for: Low-dose intravenous immunoglobulin treatment for complex regional pain syndrome (LIPS): study protocol for a randomized controlled trial
Source: Trials. 2014 Oct 24;15:404. doi: 10.1186/1745-6215-15-404 (PMC4226877; doi:10.1186/1745-6215-15-404)
Supplement: Supplementary file 5 — Additional file 5: Summary of substantial amendments. A table that details each substantial amendment that has been submitted for this protocol and what exactly was changed during that amendment. (DOCX 14 KB) [file 13063_2013_2280_MOESM5_ESM.docx]

Additional file 5 is a table that details each substantial amendment that has been submitted for this protocol and what exactly was changed during that amendment.

**Additional file 5**

Summary of substantial amendments

| **Substantial amendment**  **number** | Summary of changes |
| --- | --- |
| Substantial amendment 1, Protocol version 2.0, (19.10.12) | **Sponsor contact**  The new details reflect a change to the legal representative for one of the sponsors  **7.1 Primary outcome measure**  The new text describes an extended period for 24 hour pain diary from 15 to 37 days. An additional description of the text prompting system is described to improve compliance.  This change reverts back to the original design agreed by the funders as the TSC felt it more robust.  **7.2 Secondary outcome measures**  The EQ-5D will now be used as a measure of quality of life.  The rewording clarifies the two parts to the secondary outcomes. The secondary outcomes and the Exploratory outcomes. There were additionally missing references for the assessments to be used that have now been included. The standard gamble was removed on the advice of a health economist.  **8.2 Exclusion criteria**  Serum IgA levels previously defined as an exclusion criteria, have now been redefined.    An additional exclusion criterion has been included to prevent the inclusion of individuals that have participated in an intervention trial within the last 3 months.  **9 Screening Recruitment and consent**  Additional text has been included for clarification.  **10.3 Selection & Timing of Dose for each participant**  Additional details included to clarify the inclusion of participants that are non-compliant for the infusion visit.  **10.6 Packaging and Labelling of Investigational medicinal product**  Study name and Eudract number now included on label  **10.11 Concomitant Medications**  Additional information is provided on how to proceed if there is a change in a participant’s condition with regards to CRPS and trial intervention.  **10.12.2 Biochemistry**  Additional tests have been included for biochemistry.  **11.3 Implementation Procedures**  Clarification of the implementation procedures, including a checklist for the site nurses to go through before a participant can be randomised.  **14.1.1 Efficacy Safety**  Treatment stopping rules agreed and included.  The reporting of AE’s has also been clarified.  **14.2 Sample size calculation**  The sample size calculation was adjusted to account for the increased number of days included for the measurement of the primary outcome.  This change reverts back to the original design agreed by the funders as the TSC felt it more robust.  **15.3 Withdrawal of participants**  The following points were included to clarify discontinuation of participants in the study.  **16.2 Monitoring Quality Control and Assurance Safety**  Changes have been made to the representatives in the TSC and the DMEC  **Addition of PI video**  The video is intended to standardise the explanation of the trial across sites. As the outcome is subjective this is felt to be of importance |
| Substantial amendment 2, Protocol version 3.0, (11.04.13) | **REC**  Updated REC address  **7.2 Secondary and exploratory outcome measures**  Removal of time trade off scale.  **8.1 Inclusion criteria**  Rewording of inclusion criteria 4:  Previous specialised pain physiotherapy (24) (where not contraindicated or refused by the patient).  **10 STUDY MEDICATION**  Clarification of drug/placebo availability, drug/placebo labelling and packaging. Changes to the blinding procedure.  **10.12.3 pregnancy**  Addition of urine pregnancy test at visit 4 for females wanting open lebel drug  **Summary of study procedures**  Clarification to study procedures  **PI video**  Addition of slides |
| Substantial amendment 3, Protocol version 4.0, (01.07.13) | **7.2 Secondary outcome measures**  Neglect-Like Symptoms in CRPS questionnaire has been added to the list of measures to be used within the secondary and exploratory outcome measures  **14 STATISTICAL CONSIDERATIONS**  Primary analysis: Allergy status & low baseline IgG plasma level have been added to secondary analysis |
| Substantial amendment 4, Protocol version 5.0, (02.09.2013) | **Protocol (throughout)**  Additional site, Leicester  Minor corrective changes  **Participant Information Sheet**  Amendment to common, occasional and rare side effect of IVIG |
